# A Simple Method to Produce Recombinant Mammalian Serum Albumins in *Escherichia coli* Preserving Intact Antigenic Properties

**DOI:** 10.3390/ijms27114853

**Published:** 2026-05-28

**Authors:** Anna S. Dolgova, Anna S. Cherkashina, Alexander I. Shcherbakov, Kseniya A. Lashkevich, Irina A. Goptar, Irina P. Lisyukova, Anna E. Sudina, Olga A. Stukolova

**Affiliations:** 1 St. Petersburg Pasteur Institute, St. Petersburg 190013, Russia; 2 Central Research Institute of Epidemiology, Moscow 111123, Russia; ovasika@yandex.ru (O.A.S.); 3 Martsinovsky Institute of Medical Parasitology, Tropical and Vector-Borne Diseases, Sechenov University, Moscow 119435, Russia; 4 Federal State Budgetary Institution Centre for Strategic Planning and Management of Biomedical Health Risks of the Federal Medical Biological Agency, Moscow 119435, Russia

**Keywords:** serum albumin, canine serum albumin, Can f 3, feline serum albumin, Fel d 2, microarray, antigenic properties, *E. coli*, mammalian protein expression

## Abstract

Serum albumin (SA) plays a fundamental role in the transport of metabolites and endogenous ligands. Additionally, animal albumins are potent allergens. Heterologous expression of SAs is challenging due to their complex structure. In this study, we describe a simple method for enhanced production of soluble and functional recombinant feline (rFel d 2) and canine (rCan f 3) albumins in the standard *E. coli* strain BL21 (DE3). To achieve this, the 18-amino acid signal peptide was removed from the N-terminus of each albumin. To improve expression, folding, and solubility of recombinant proteins, we tested an extended panel of fusion proteins. Among them, MBP (maltose-binding protein), TF (trigger factor), and NusA (*E. coli* transcription termination factor) allowed the production of soluble fusion forms of rFel d 2 and rCan f 3. To confirm the structural integrity of the products, we analyzed the IgE-binding characteristics of recombinant versus native albumins. rFel d 2 and rCan f 3 fused with TF, MBP, and NusA bound albumin-specific immunoglobulin E (in 26/33, 29/33, 31/33, and 27/29, 26/29, 27/29 cases, respectively, comparable to native Fel d 2 and Can f 3. Thus, removal of the signal peptide combined with fusion partners enables expression of Fel d 2 and Can f 3 in *E. coli* with preserved antigenic properties.

## 1. Introduction

The most widespread and sought-after method for expressing recombinant proteins is the bacterial system based on *Escherichia coli* (*E. coli*) due to its relatively low cost, ease of use, productivity, and scalability [1]. However, when it comes to eukaryotic proteins, which often have multidomain organization, various post-translational modifications, disulfide bonds, and complex spatial structures, efficient production in bacterial cells is often challenging and requires specialized expression strains and optimized conditions.

Serum albumins (SAs) have high concentrations in blood plasma and serve as vascular transporters for hormones, free radicals, metal ions, fatty acids, sugars, and drugs [2]. Due to their abundance and biological importance, SAs have wide clinical and research applications [3,4,5].

SAs are highly conserved both in amino acid sequence and three-dimensional structure. Despite the fact that the amino acid sequences of mammalian and human serum albumins are more than 80% identical, some people develop allergic reactions. Up to 40% of patients who are allergic to animal dander exhibited immunoglobulin E (IgE) that is reactive towards serum albumins [6,7,8,9]. Thus, animal albumins are becoming increasingly relevant in allergy studies, diagnostics, treatment, and prevention. The most frequently used in diagnostics albumins are horse Equ c 3, guinea pig Cav p 4, cat Fel d 2, and dog Can f 3. Sensitization to Fel d 2 and Can f 3 is of particular interest as these allergens originate from the most common household pets. To date, modern molecular-based allergy diagnostic kits use the individual allergens that are either isolated directly from the allergen source or produced recombinantly, which is easier and more profitable.

Animal albumins are primarily obtained from animal blood. Even human serum albumin (HSA), widely used therapeutically outside allergology, is mostly sourced from human plasma because it forms inclusion bodies in bacterial and yeast expression systems, while production in eukaryotic cell cultures is difficult and expensive [10].

Recombinant human serum albumin has been successfully produced in *S. cerevisiae*, *K. lactis*, and *P. pastoris* expression systems, even for pharmaceutical purposes [11]. However, attempts to produce recombinant albumins in the more common and easy-to-use *E. coli* system cannot yet be considered fully successful.

The main challenges arise from the protein’s large size (65–69 kDa), multidomain organization, and the presence of 17 conserved disulfide bonds. Overexpressed inactive forms of SA tend to aggregate and densely pack into inclusion bodies [12,13].

Recombinant canine and feline albumins, Can f 3 and Fel d 2, were predominantly expressed in *E. coli* in insoluble form. Nevertheless, following resolubilization and purification, these proteins demonstrated the capacity to specifically bind native albumin-specific IgE (sIgE) present in serum samples from patients allergic to cats or dogs [14,15]. However, the isolation of proteins from inclusion bodies is inherently laborious, resource-intensive, and characterized by low overall yield, rendering soluble protein expression a more desirable objective.

Successful expression of the soluble form of HSA in *E. coli* has been accomplished via fusion with solubility-enhancing partners such as maltose-binding protein (MBP) and protein disulfide isomerase b’a’ domain (PDIb’a’) [16]. Prior studies have also reported modest but notable yields of soluble recombinant HSA through coexpression with molecular chaperones and optimization of expression parameters [17]. Additionally, targeted mutagenesis of the protein sequence has been explored as a strategy to improve soluble form expression levels [18].

SAs possess at least one conformational epitope alongside linear epitopes [19], and only SAs with intact disulfide-bond stabilized tertiary structure can effectively bind with sIgE [20]. Consequently, assessment of sIgE reactivity serves as an efficient proxy for verifying the structural integrity of recombinant albumins, obviating the need for more costly and time-consuming three-dimensional structural analyses.

Within this context, the development of a streamlined and efficient method for the rapid production of recombinant albumins in a soluble, native-like conformation represents a significant advance not only for diagnostic applications but also for structural and epitope mapping studies, investigation of molecular transport mechanisms, drug discovery efforts, and analyses of antibody cross-reactivity. Herein, we present a rapid and straightforward approach for obtaining recombinant feline and canine albumins using the standard *E. coli* BL21 (DE3) strain, with subsequent confirmation of their antigenic properties.

## 2. Results

### 2.1. Expression Cassette Development and Recombinant Protein Production

The first 18 amino acids at the N-terminus of the sequences of canine and feline albumins taken from GenBank were identified as signal peptides (SPs): mkwvtfisllllfssays for Fel d 2 and mkwvtfislfflfssays for Can f 3. For expression in *E. coli*, sequences encoding these peptides were excluded. The resulting sequences without signal peptides were named dsFel d 2 and dsCan f 3 (ds = deleted signal peptide). The resulting DNA sequences were submitted to GenBank under accession numbers ON646010 and ON646009 for dsFel d 2 and dsCan f 3, respectively.

**Figure 1:**
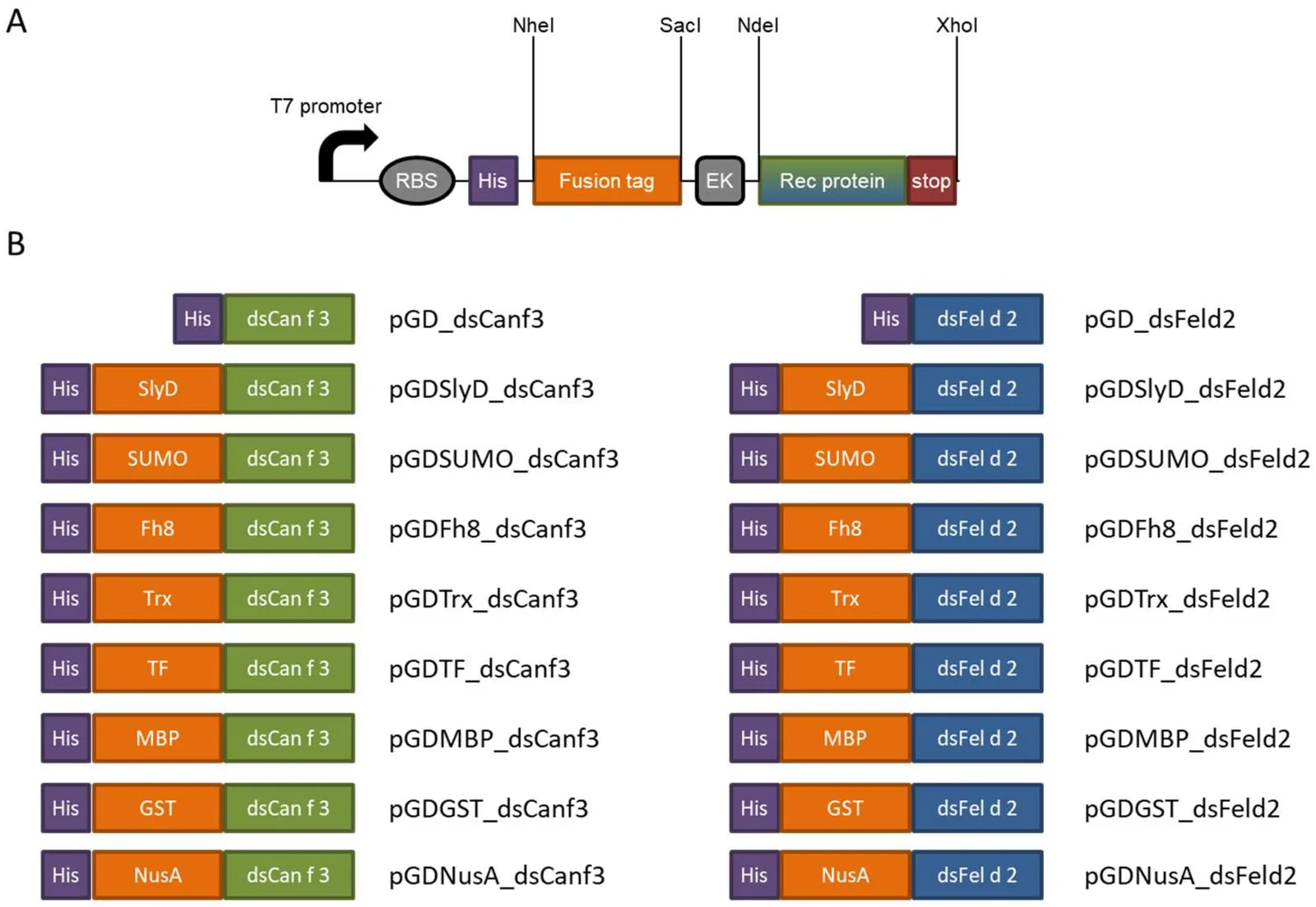
Schematic graph of constructed plasmids (**A**) and synthesized proteins (**B**). His—6× histidine tag; SlyD—prolyl cis/trans isomerase; SUMO—small ubiquitin-like modifier protein; Fh8—protease from the liver fluke Fasciola hepatica; Trx—thioredoxin; TF—trigger factor; MBP—maltose-binding protein; GST—glutathione-S-transferase; NusA—*E. coli* transcription termination factor; dsFel d 2—feline albumin without the N-terminal 18-amino-acid signal peptide; dsCan f 3—canine albumin without the N-terminal 18-amino-acid signal peptide; pGD—pET24-based expression vector with a customized polylinker sequence; EK—enterokinase cleavage site.

To streamline cloning of six fusion tags with two target proteins, universal backbones based on the pGD plasmid (Figure 1A) were first designed and prepared. These backbones were then used to construct expression vectors. Consequently, dsFel d 2 and dsCan f 3 were fused with prolyl cis/trans isomerase (SlyD, 21 kDa); small ubiquitin-like modifier protein (SUMO, 11 kDa); *Fasciola hepatica* liver protease (Fh8, 8 kDa); thioredoxin (Trx, 12 kDa); trigger factor (TF, 48 kDa); maltose-binding protein (MBP, 42 kDa); glutathione-S-transferase (GST, 26 kDa); and *E. coli* transcription termination factor (NusA, 55 kDa). The corresponding plasmids and illustrated fusion proteins are listed in Figure 1B. The enterokinase cleavage site (EK) has been added to the expression cassette sequence, but splitting was not performed during this study.

**Figure 2:**
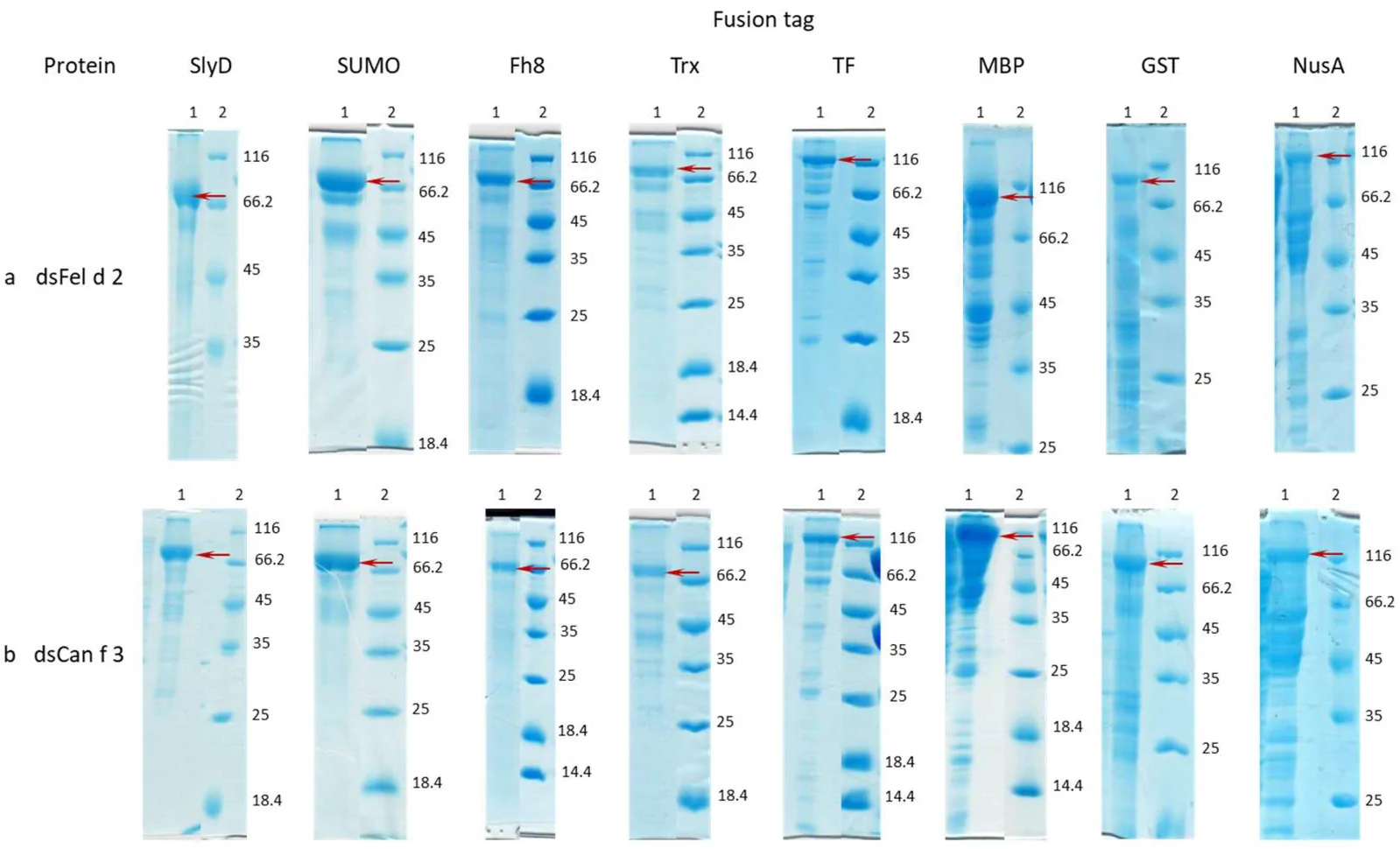
SDS-PAGE of recombinant dsFel d 2 (**a**) and dsCan f 3 (**b**) fractions used for further analysis. The corresponding band is compiled with the molecular weight markers band from the same gel, and arrows indicate the target product.

The resulting expression vectors were transformed into the standard *E. coli* strain BL21 (DE3) for protein production. Recombinant proteins were purified by metal affinity chromatography using nickel-activated Sepharose. Protein fractions were selected for further analysis based on sodium dodecyl sulfate–polyacrylamide gel electrophoresis (SDS-PAGE) results (Figure 2 and Supplement S2) and are listed in Table 1, Tables S1 and S2, Supplement S1, and Figure S1. Fusion tags were not removed prior to IgE-binding assays.

dsFel d 2 and dsCan f 3 proteins expressed as fusions with TF, MBP, and NusA tags were obtained in soluble form from *E.* coli cell lysates. dsFel d 2 and dsCan f 3 proteins expression as fusions with SlyD, SUMO, Fh8, Trx, and GST tags did not result in a significant amount of soluble form. Thus, these proteins were extracted from *E. coli* cell debris under denaturation conditions. Expression of rCan f 3 with the signal peptide did not yield detectable protein levels (Supplement S2). Concurrently, cat serum albumin (nFel d 2) and native dog serum albumin (nCan f 3) were isolated from pooled animal blood plasma for subsequent comparative analysis with the recombinant proteins (Supplement S2).

### 2.2. Microarray

192 serum samples from patients with confirmed sensitization to cats (plus 71 negative controls) and 187 samples from patients sensitized to dogs (plus 58 negative controls) were analyzed. sIgE against nFel d and nCan f 3 was detected in 33 (17.2%, 95% CI 12.1–23.3) and 29 (15.5%, 95% CI 10.6–21.5) samples, respectively (Table 2).

The comparison of recombinant proteins with native proteins in albumin-positive cohorts is summarized in Table 3. For Fel d 2, the highest sensitivity was observed for TF (26/33), MBP (29/33), and NusA (31/33) fusion constructs; for Can f 3, the best results were achieved with MBP (26/29), TF (27/29), and NusA (27/29). Recombinant proteins lacking fusion tags showed considerably lower sensitivity compared to their native counterparts.

Of all tested recombinant proteins across both positive and negative sample cohorts, false positive responses compared to ImmunoCAP results were observed in 1 sample for nFel d 2, in 1 sample for TRX_dsFel d 2, and in 2 samples for TRX_dsCan f 3. No false positives were detected in the control cohort with other antigens. Within ImmunoCAP-positive cohorts, some samples were negative for nFel d 2 or nCan f 3 but positive for certain recombinant albumin fusion variants. Specifically, one sample was positive for both TRX_dsFel d 2 and SlyD_dsFel d 2; three samples were positive only for NusA_dsFel d 2; four samples were individually positive for TRX_dsCan f 3; one sample was positive exclusively for TF_dsCan f 3; and one sample was simultaneously positive for TF, MBP, and NusA_dsCan f 3. Based on these findings, sensitivity and specificity values were calculated, as presented in Table S2 in Supplement S1 Table S4.

Thus, during this study, we have demonstrated that removal of the signal sequence enabled the production of recombinant albumin in *E. coli* capable of binding specific anti-albumin antibodies in the blood serum of patients with confirmed sensitization to cat or dog fur and dander. Furthermore, incorporating TF, MBP, and NusA fusion tags enhanced the sensitivity of the microarray assay.

## 3. Discussion

The main goal of this study was to obtain recombinant animal serum albumins, which are valuable for diagnostic and research purposes. We focused on feline and canine albumins, Fel d 2 and Can f 3, known as common allergens originating from the most widespread household pets.

There is growing interest in personalized care based on molecular, immunologic, and functional endotyping, particularly in molecular-based allergy diagnostics, now referred to as precision allergy molecular diagnostic applications (PAMD@) [21]. Nowadays, the most advanced diagnostic systems are the ImmunoCAP™ ISAC assay and ALEX [9,22,23,24]. These platforms include individual allergenic molecules for in vitro sIgE testing, incorporating Fel d 2 and Can f 3 among them.

To date, animal serum albumins used in molecular-based allergy diagnostics are primarily obtained from animal blood. It is worth noting the use of recombinant Fel d 2 in the ImmunoCAP™ ISAC assay [25], although the expression system employed is not disclosed.

In our study, sIgE to nFel d 2 and nCan f 3 were detected in 33 (17.2%, 95% CI 12.1–23.3) and 29 (15.5%, 95% CI 10.6–21.5) samples, respectively (Table 2). These values are consistent with existing statistics, considering that albumins are not the predominant animal allergens. Approximately 15–25% of cat-allergic patients are sensitized to feline serum albumin [26]. Can f 3 binds specific IgE in 10–20% of dog-sensitized patients [27,28].

Fel d 2 has reportedly been expressed in yeast cells [29,30], but this method requires long-term induction, which increases the risk of protein degradation due to endogenous and secreted peptidase activity.

Expression of Fel d 2 and Can f 3 in *E. coli* typically results in accumulation of the proteins in inclusion bodies [14,15]. Protein solubilization and refolding from inclusion bodies are labor-intensive and tend to yield low recovery, making this approach less favorable. Obtaining these proteins in a soluble form is more efficient, often achieved using fusion protein strategies. Various fusion tags are commonly employed to promote proper protein folding, protect against degradation, enhance recombinant protein expression, and facilitate purification and detection [24,31,32].

Additional optimization strategies include adjusting culture conditions (temperature, shaking speed, and additives), co-expression with molecular chaperones, using engineered *E. coli* strains, codon optimization, and introducing targeted mutations to improve protein solubility and stability.

Expression of albumins in the presence of a signal peptide did not yield detectable protein levels (Supplement S2). The data indicate that removal of these peptides enables protein expression, suggesting that the signal peptides interfered with translation. Translocation across the ER membrane has evolved from translocation across the plasma membrane in prokaryotes, but it is not the same. It is known that some eukaryotic signal peptides can impair expression in prokaryotes or even be toxic to *E. coli* [33,34].

Recombinant Can f 3 was successfully produced in *E. coli* and demonstrated sIgE binding, although no comparative sensitivity analysis between recombinant and natural Can f 3 was performed. This success was achieved by using a template mRNA and a 5′ primer that amplified DNA starting at the 28th lysine residue, thus enabling expression without the N-terminus and excluding the signal peptide sequence [14].

The SPs for Fel d 2 (mkwvtfisllllfssays) and Can f 3 (mkwvtfislfflfssays), predicted by SignalP, are typical secretory peptides transported via the Sec translocon and cleaved by Signal Peptidase I (Lep) (Sec/SPI), mediating extracellular localization [35]. These albumin signal peptides are highly conserved; for example, the human albumin SP efficiently directs protein secretion with very high efficiency in cells derived from different mammals [36]. This conservation implies functional interchangeability of these signal peptides among mammals.

Other major limitations associated with the production of recombinant mammalian proteins in bacterial systems include the tendency of overexpressed proteins to aggregate and form dense, inactive structures known as inclusion bodies [13]. Because these proteins fail to fold correctly in the *E. coli* cytoplasm, optimization of in vitro refolding protocols combined with denaturation and purification steps is often required to recover active products. This process significantly increases production costs and reduces overall protein yield [13,37].

The fusion protein strategy has proven effective in addressing several challenges within the *E. coli* expression system. It facilitates proper protein folding, protects recombinant proteins from proteolytic degradation, enhances expression levels, and simplifies both purification and detection procedures [24,31].

The combination of MBP and PDIb’a’ tags, the *E. coli* Origami 2 strain, and low induction temperature led to a successful expression and purification of HSA in soluble form from *E. coli* [16]. In our current work, we adopted a similar strategy by designing a set of expression vectors combining various fusion tags with target albumins. We tested several popular tags, including those previously effective with HSA.

SlyD is a bacterial two-domain protein acting as a molecular chaperone, prolyl cis/trans isomerase, and nickel-binding protein. Prolyl isomerases are involved in a broad range of biological functions, and in particular, they assist protein folding, thereby improving the solubility of fused proteins [38,39]. SUMO fusion enhances protein expression, solubility, and purification in prokaryotic hosts and is widely used [34,40,41]. Fh8, an 8 kDa protease from *Fasciola hepatica*, is a low molecular weight tag effective at enhancing solubility [42,43,44]. Trx provides stability and high solubility to fused polypeptides that might otherwise aggregate in bacterial host cells. It is an efficient fusion tag for human and endogenous redox-active proteins, facilitating correct disulfide (S–S) bond formation [45,46,47,48,49]. TF is a 48 kDa prokaryotic ribosome-associated chaperone that promotes cotranslational folding, reducing misfolding and insolubility. Its *E. coli* origin results in high expression levels in *E. coli* systems [50,51,52]. MBP is one of the most popular fusion partners for bacterial expression, enhancing solubility and playing a passive role in folding [53,54]. GST, a highly expressed eukaryotic protein, promotes solubility and expression as an N-terminal fusion. It is among the earliest affinity tags (since 1988) and remains widely used for solubility enhancement [55,56,57]. NusA improves soluble protein production with a passive folding role similar to MBP [24,54,58]. This comprehensive use of various fusion tags leverages their distinct biochemical properties—chaperone activity, folding assistance, solubility enhancement, and stabilization—to increase soluble recombinant protein yield in *E. coli* expression systems.

In our study, the highest yield of soluble recombinant SAs was achieved when the MBP tag was used (22.56 and 45.46 mg/L for MBP-dsFel d 2 and MBP-ds Can f3, respectively), followed by TF and NusA proteins (Table 1). High expression of rSAs in *E.coli* with MBP tag was reported previously with similar soluble product yield [16]. The main difference was that in our study, the widely used *E.coli* strain was utilized due to expression conditions optimization.

Cat and dog SAs are not the main object for recombinant SA studies, most of which are focused on rHSA efficient expression. Currently, the rHSA reached clinical trials utilize yeast expression such as *Pichia pastoris* (*Komagataella phaffii*), allowing for the achievement of yield from approximately 1 g/L to 17.5 g/L rHSA with the product exhibiting high physicochemical similarity to native HSA [59,60]. But the O-glycosylation modification during yeast expression may impact product stability and immunogenicity [61]. Also, the cultivation process takes up to a week, requires special temperature conditions, and enrichment of the culture medium. The cost of purified yeast-derived rHSA is estimated to be around 2.34–3.22 USD/gram if large-scale production is used [61].

The transgenic plant expression system may have low cultivation costs, simple medium requirements, and facile scalability for large-scale production [62]. Expression level of rHSA in transgenic rice seeds was around 2.75 g of high-purity product per kilogram [63], 30 μg/g fresh leaf weight in the *Nicotiana benthamiana* [64], and 33.92 μg/g fresh weight in purslane [65]. Separation and purification processes of plant-produced rHSA need further optimization, and plant-specific glycosylation patterns may affect protein homogeneity and functionality [61]. The cultivation process in this case takes up to six months, which is inconvenient and expensive for research purposes. However, while using it in manufacturing, the final cost drops to 1–1.9 USD/g [61].

Mammalian cell expression systems can fulfill precise glycosylation, carboxylation, and amidation, which are most similar to human modifications. CHO and HEK293 cells have been used to express biologically active HSA [66,67,68]. The mammalian cell expression of rSAs does not seem to have wide distribution now or in the future, as the cost of such cultivation is high, the length of the production cycle is up to 2 weeks, and the protein yield is low.

Unfortunately, no methods can unambiguously predict the effect of a fusion tag on a target protein, so the optimal fusion must still be determined empirically. According to our data, fusions of feline and canine albumins Fel d 2 and Can f 3 with TF, MBP, and NusA enabled production of recombinant proteins in soluble form in *E. coli*. Additionally, these fusions, along with SlyD, allowed the recombinant proteins to react appropriately with sIgE antibodies.

The absence of nonspecific reactions—from both tags and non-optimal purification processes—was confirmed using a panel of negative control samples. No unspecific binding was noticed even if the purity of the used protein fraction didn’t exceed 80%.

More specifically, NusA fusions yielded the best results. Fusion of dsFel d 2 with NusA detected 93.9% (31/33) of positive samples compared to native albumin (Table 2), enabling identification of additional positive sera within the ImmunoCAP-positive cohort, while maintaining sensitivity and specificity comparable to the native protein (Supplement S1 Table S4). Similarly, NusA fusion to dsCan f 3 demonstrated 93.1% sensitivity compared to native Can f 3.

MBP fusions also showed high sensitivity: dsFel d 2 and dsCan f 3 fusions with MBP detected 87.8% and 89.7% of positive samples, respectively, compared to native analogs. TF fusion is a promising candidate as well. The TF-dsCan f 3 fusion identified 27/29 positive to nCan f 3 albumin samples (93.1%), plus two additional ImmunoCAP-positive samples, maintaining overall sensitivity. However, TF-dsFel d 2 fusion detected only 26/33 nFel d 2—positive samples (78.8% sensitivity), which is notably lower. This discrepancy likely reflects differences in protein purity, as TF-dsFel d 2 preparations were slightly less pure. Since high purity of obtained fusion recombinant proteins was not the study’s primary objective, TF fusion remains a viable option for recombinant albumin production. As all NusA, TF, and MBP are relatively big proteins, some steric constraints could influence the sIE binding. This is the point of future investigation using EK disintegration of fusion tag and rSAs.

We did not assess the exact conformation of the obtained proteins, as this was beyond the scope of the study. More importantly, we clearly demonstrate that the current conformation of recombinant albumins fused to defined tags is sufficient for recognition of epitopes by specific antibodies with high specificity and sensitivity, comparable to native albumins. In other words, irrespective of the recombinant protein’s final conformation, it is possible to select a fusion that allows antibody recognition, indicating preservation of both linear and conformational epitopes in this complex, multi-domain globular protein [20].

## 4. Materials and Methods

### 4.1. Patients and Serum Samples

Serum samples were collected with informed consent from participants for anonymous use in routine investigations in the clinic diagnostic laboratory at the Center of Molecular Diagnostics, Federal Budget Institution of Science ‘Central Research Institute of Epidemiology’ (CMD CRIE) under the Federal Service for Surveillance on Consumer Rights Protection and Human Wellbeing, Moscow, Russia. Subjects in the allergic cohorts exhibited specific IgE levels ≥ 0.35 IU/mL to cat dander extract (e1) (*n* = 192) or to dog epithelium or dander extracts (e2 or e5) (*n* = 187), as determined by the ImmunoCAP (Phadia, Thermo Fisher Scientific, Waltham, MA, USA). The mean age of the cat IgE-positive group was 19.3 years, while the mean age of the dog IgE-positive group was 20.7 years.

Serum samples from 71 individuals (mean age 21.6 years) and from 58 individuals (mean age 17.4 years) with ImmunoCAP scores of 0 for e1 or for e2 and e5, respectively, were used as negative control groups.

### 4.2. Gene Synthesis and Cloning

Protein sequences of Fel d 2 and Can f 3 were taken from GenBank (accession numbers CAA59279.1 and BAC10663.1, respectively) and are referenced at http://www.allergen.org/. Signal peptides were predicted using the SignalP server (version 6.0) [35] and subsequently removed from the coding sequences. The following fusion tags were employed: SlyD, SUMO, Fh8, Trx, TF, MBP, GST, NusA, and 55 kDa. Fusion tag sequences were obtained from GenBank (accession numbers CAD6001205.1, NP_010798.1, AF213970.1, WP_194499549.1, WP_205899502.1, WP_247154701.1, WP_164689877.1, and WP_021518520.1 for SlyD, SUMO, Fh8, Trx, TF, MBP, GST, and NusA, respectively) and customized. The resulting fusion tag amino acid sequences are listed in Table S1, Supplement S1, Figure S1.

The coding DNA sequences were designed and synthesized de novo as described previously [69]. Genes were cloned into the pGEM-T Easy vector (Promega, Madison, WI, USA) and sequenced using Applied Biosystems 3500 Series Genetic Analyzers (Thermo Fisher Scientific, USA).

For protein expression, a pGD vector series based on pET24a was used, where the sequence between the XbaI and XhoI restriction sites was replaced (Supplement Figure S1). Each expression plasmid contained an N-terminal 6xHis-tag, the fusion protein, and either Fel d 2 or Can f 3. Recombinant plasmids were named as follows:

- For Fel d 2: pGDSlyD_dsFeld2, pGDSUMO_dsFeld2, pGDFh8_dsFeld2, pGDTrx_dsFeld2, pGDTF_dsFeld2, pGDMBP_dsFeld2, pGDGST_dsFeld2, pGDNusA_dsFeld2, and pGD_dsFeld2.
- For Can f 3: pGDSlyD_dsCanf3, pGDSUMO_dsCanf3, pGDFh8_dsCanf3, pGDTrx_dsCanf3, pGDTF_dsCanf3, pGDMBP_dsCanf3, pGDGST_dsCanf3, pGDNusA_dsCanf3, pGD_dsCanf3, and pGD_Canf3.

Plasmids pGD_dsFeld2, pGD_dsCanf3, and pGD_Canf3 contained only the 6xHis-tag without additional fusion tags.

### 4.3. Protein Expression

Different combinations of seeding, reseeding, and cultivation conditions were tested for each expression vector. The conditions yielding the highest protein expression are summarized in Table S2, Supplement S1, and Figure S1. In each case, 100 µL of *E. coli* BL21 (DE3) competent cell culture was heat-shock transformed with the expression plasmids and seeded onto LB agar Petri dishes containing appropriate additives (Table S2, Supplement S1, Figure S1). Petri dishes were incubated overnight at 35 °C. Colonies were then harvested from the LB agar with 1.0 mL of LB medium. Glycerol was added to a final concentration of 10% to prepare *E. coli* cell suspensions. Seed cultures were aliquoted and stored at −70 °C for no longer than six months. Reseeding on solid media was performed to increase the amount of seed material immediately prior to cultivation (Table S2, Supplement S1, Figure S1).

Subsequently, *E. coli* cells were washed off the solid media into 2 L of LB medium supplemented with 50 µg/mL kanamycin and other additives (Table S2, Supplement S1, Figure S1). Cultivation was performed either overnight or during the day (Table S2, Supplement S1, Figure S1). Overnight cultivation was carried out in an orbital shaker at 220 rpm and 30 °C in LB medium containing 50 µg/mL kanamycin, 2.5% glycerol, and 50 mM isopropyl β-D-1-thiogalactopyranoside (IPTG) for continuous induction of protein expression. Daytime cultivation proceeded in two stages: first, pre-induction growth for 2–3 h at 35 °C with shaking at 220 rpm in LB medium supplemented with 50 µg/mL kanamycin, 1% glucose, and 1–2% glycerol; second, cultures were cooled to 30 °C, IPTG was added to a final concentration of 0.5 mM, and cultivation continued for 4 h under the same shaking and temperature conditions.

Following overnight or daytime cultivation, cultures were centrifuged at 3000× *g* and 4 °C for 15 min. Cell pellets were resuspended in 100 mL of 50 mM Tris/HCl buffer (pH 8.0) containing 1 mg/mL lysozyme. After complete dissociation of cell aggregates, 100 mL of lysis buffer (50 mM Tris/HCl, pH 8.0, containing 1 M NaCl, 1% Triton X-100, 0.4% Tween-20) was added, and cells were disrupted by ultrasonication for 150 s at 15 °C. The resulting lysates were centrifuged at 10,000× *g* and 4 °C for 30 min. Supernatants were subjected to affinity chromatography purification. The pellet containing cell debris was extracted with 100 mL of 50 mM Tris/HCl buffer (pH 8.0) containing 6 M urea and 0.5 M NaCl, followed by centrifugation at 10,000× *g* and 4 °C for 30 min. The resulting supernatants were also purified by affinity chromatography.

### 4.4. Chromatography on Ni-Activated Sepharose

Two hundred milliliters of the obtained cell lysates and the soluble fraction of cell debris were purified by affinity chromatography using Ni-activated Chelating Sepharose™ Fast Flow resin (Cytiva, Marlborough, MA, USA), prepared according to the manufacturer’s instructions. Briefly, 8 mL of 50 mM NiCl_2_ solution was passed through 8 mL of Chelating Sepharose™ Fast Flow resin. Subsequently, 50 mL of purified water was passed through the Ni-activated resin, followed by 50 mL of starting buffer. The starting buffer for lysate purification consisted of 50 mM Tris/HCl (pH 8.0) with 0.25 M NaCl, whereas for purification of the soluble fraction of debris, 50 mM Tris/HCl (pH 8.0) with 0.2 M NaCl and 6 M urea was used.

Cell lysates or soluble debris fractions were then loaded onto the resin, followed by washing with 50 mL of the starting buffer. Elution was performed using 50 mM Tris/HCl (pH 8.0) containing 0.2 M NaCl and imidazole at concentrations of 0.06, 0.3, or 1 M for lysate purification, and similarly for the soluble debris fraction with the addition of 6 M urea. All eluted fractions were stored at +4 °C.

After finishing each chromatography, the column was washed sequentially with 15 mL of 100 mM EDTA with 0.5 M NaCl, purified water, 2 M NaCl, purified water, 1 M NaOH, purified water, and finally 2 M NaCl, followed by purified water.

### 4.5. Native Albumins Purification

nFel d 2 and nCan f 3 were purified as described previously [70] from pooled cat or dog blood plasma, respectively (a detailed protocol is provided in Supplement S1, Figure S1).

### 4.6. SDS—PAGE and Protein Concentrations Determination

Fractions of native albumins, lysates, supernatants, and pellets obtained after dissolution of *E. coli* cell debris, unbound material, and imidazole elution fractions were analyzed by reducing SDS—PAGE using 12% separating and 4% stacking polyacrylamide gels stained with Coomassie G250 (Supplement S2). Optical density at 280 nm was measured in the obtained fractions, and protein concentrations were calculated using an extinction coefficient of 1.0 (Table S3, Supplement S1, Figure S1). Additionally, the purity of obtained proteins was estimated using GelDoc Go Gel Imaging System with Image Lab Touch Software (Bio-Rad, Hercules, CA, USA).

### 4.7. Microarray Preparation

The protein array preparation, procedure, and readouts were performed as described in detail in Supplement S1, Methods S2, and elsewhere [8].

In brief, solutions of selected fractions of native and recombinant proteins, along with negative and positive controls (anti-human IgE), were spotted onto the bottoms of 96-well plates using piezoelectric printing (SX, Scienion AG, Dortmund, Germany) to produce IFA-like tests in a microarray format. The next steps included incubation with diluted serum samples (from patients with predefined positive or negative IgE responses by ImmunoCAP), washes, incubation with biotin-conjugated anti-human IgE antibodies, incubation with Cy5-conjugated streptavidin, and fluorescence detection. The median fluorescence signal obtained from spots containing each protein was used to calculate cutoffs and determine the presence of specific antibodies against each protein.

### 4.8. Statistics

Analyses were performed using MedCalc v20.027 (MedCalc Software Ltd., Ostend, Belgium). The study cohorts were stratified into two groups: (i) patients with a positive IgE reaction in ImmunoCAP (e1 or e2/e5) and (ii) patients with a negative IgE reaction in ImmunoCAP (e1 or e2/e5). ROC analyses were conducted to determine the cut-off values. A *p*-value < 0.05 was considered statistically significant (Supplement S1, Table S4). In this section, where applicable, authors are required to disclose details of how generative artificial intelligence (GenAI) has been used in this paper (e.g., to generate text, data, or graphics, or to assist in study design, data collection, analysis, or interpretation). The use of GenAI for superficial text editing (e.g., grammar, spelling, punctuation, and formatting) does not need to be declared.

## 5. Conclusions

During our study, we developed and successfully implemented a method for generating recombinant proteins using tag protein genes and an expression vector system. This approach allowed us to produce recombinant feline and canine albumins (rFel d2 and rCan f3) in a soluble form using a standard *E. coli* strain.

Empirical testing was essential to select the optimal fusion tags for our study. NusA showed superior performance during the immunoreactivity assay, closely followed by MBP and TF. MBP yielded the highest amount of soluble product, while TF produced the highest purity of the soluble form.

Storage experiments showed that all four soluble variants (with SlyD, TF, MBP, and NusA) remained stable in elution buffer at +4 °C for up to 12 months without any loss of specific antibody-binding activity for each albumin.

The conformational studies were not carried out during this work. In addition, purification steps other than Ni-affinity chromatography were not performed, so the purity of the obtained products was not ideal. Furthermore, despite the inclusion of the EK site, the fusion tags were not removed prior to the testing procedures. Nevertheless, the resulting fusions of rFel d2 and rCan f3 with NusA, MBP, and TF showed high specificity and sensitivity in IgE binding.

We believe that this approach is effective and sufficient for the screening of fusions in order to obtain desired complex proteins in a soluble recombinant form, with the potential to bind corresponding antibodies in diagnostic immunoassays such as microarrays, blots, point-of-care tests, or ELISA. Additionally, these products could be used in epitope mapping, cross-reactivity, and structural studies. Since the NusA fusion was the most successful for both albumins, it may be possible to use it for the production of recombinant human or other mammalian albumins, as well as other difficult-to-express eukaryotic or viral proteins.

## Figures and Tables

**Table 1:** Fractions of recombinant cat and dog albumin fusions were used.

Protein	Fusion Tag	Form, Selected Fraction	Expected MW, kDa	Concentration ^1^ (mg/mL)	Purity ^1^, %	Soluble Form ^1^, %	Yield of Used Fraction, mg/L
dsFel d 2	-	Unsoluble, A ^2^	70	1.00	90	0	5.25
	SlyD		91	0.8	90	0	12.65
	SUMO		81	0.99	90	5	6.93
	Fh8		77	0.32	95	5	2.08
	Trx		81	0.38	90	0	2.85
	TF	Soluble, B	118	1.28	90	60	9.6
	MBP		110	3.76	80	30	22.56
	GST	Unsoluble, C	95	0.44	95	10	1.98
	NusA	Soluble, B	124	1.86	80	40	8.37
dsCan f 3	-	Unsoluble, A	70	0.62	95	0	2.48
	SlyD		91	0.92	95	0	5.52
	SUMO		81	1.25	90	5	9.38
	Fh8		77	0.28	95	0	1.54
	Trx		81	0.39	90	0	2.73
	TF	Soluble, B	119	1.77	90	60	13.27
	MBP		110	6.27	80	50	45.46
	GST	Unsoluble, A	95	2.75	80	10	20.63
	NusA	Soluble, B	124	0.62	80	70	3.10

^1^ established from SDS-PAGE. ^2^ A—50 mM Tris/HCl, pH 8.0, 0.2 M NaCl, 0.3 M imidazole, 6 M Urea; B—50 mM Tris/HCl, pH 8.0, 0.2 M NaCl, 0.3 M imidazole; C—50 mM Tris/HCl, pH 8.0, 0.2 M NaCl, 1 M imidazole, 6 M Urea.

**Table 2:** Diagnostic accuracy of native and recombinant cat and dog albumins with TF, MBP, SlyD, and NusA fusions.

Protein	Sensitivity	95% CI ^1^	Specificity	95% CI
nFel d2	17.2	12.1–23.3	98.6	92.4–100
SlyD_Fel d2 ^2^	13.5	9.0–19.2	100	94.9–100
TF_Fel d2	13.5	9.0–19.2	100	94.9–100
MBP_Fel d2	15.1	10.4–21.0	100	94.9–100
NusA_Fel d2	17.7	12.6–23.9	100	94.9–100
nCan_f3	15.5	10.6–21.5	100	93.8–100
SlyD_Can_f3	12.8	8.4–18.5	100	93.8–100
TF_Can_f3	15.5	10.6–21.5	100	93.8–100
MBP_Can_f3	14.0	9.3–19.8	100	93.8–100
NusA_Can_f3	14.4	9.7–20.3	100	93.8–100

^1^ CI—confidential interval. ^2^ Data for other proteins are presented in Supplement S1, Table S4.

**Table 3:** Comparison of recombinant proteins with native proteins.

Fusion	Protein			
	dsFel d2		dsCan f3	
	rec/nat ^1^	%, (95% CI) ^2^	rec/nat	%, (95% CI ^3^)
no	21/33	63.6 (39.4–97.3)	12/29	41.4 (21.4–72.3)
SlyD	25/33	75.8 (49.0–100.0)	24/29	82.8 (53.0–100.0)
SUMO	19/33	57.6 (34.7–89.9)	15/29	51.7 (28.9–85.9)
Fh8	16/33	48.5 (27.7–78.7)	10/29	34.5 (16.5–63.4)
TRX	11/33	33.3 (16.6–59.6)	18/29	62.1 (36.8–98.1)
TF	26/33	78.8 (51.5–100.0)	27/29	93.1 (61.4–100.0)
MBP	29/33	87.9 (58.9–100.0)	26/29	89.7 (58.6–100.0)
GTS	22/33	66.7 (41.8–100.0)	13/29	44.8 (23.9–76.7)
NusA	31/33	93.9 (63.8–100.0)	27/29	93.1 (61.4–100.0)

^1^ rec/nat—the ratio of the number of IgE-positive samples for the recombinant protein to the number of samples positive for the native protein. ^2^ %—the ratio of the number of IgE-positive samples for the recombinant protein to the number of samples positive for the native protein as a percentage. ^3^ CI—confidential interval.

## Data Availability

All sequences that support the findings of this study have been deposited in GenBank. Further information is available from the corresponding author upon reasonable request.

## Supplementary Materials

The following supporting information can be downloaded at: https://www.mdpi.com/article/10.3390/ijms27114853/s1.

## Abbreviations

The following abbreviations are used in this manuscript:

*E. coli*	*Escherichia coli*
SA	Serum albumin
IgE	Immunoglobulin E
Equ c 3	Horse serum albumin
Cav p 4	Guinea pig serum albumin
Fel d 2	Cat serum albumin
Can f 3	Dog serum albumin
HSA	Human serum albumin
MBP	Maltose-binding protein
PDIb’a’	Protein disulfide isomerase b’a’ domain
SP	Signal peptide
SlyD	prolyl cis/trans isomerase
SUMO	small ubiquitin-like modifier protein
Fh8	*Fasciola hepatica* liver protease
Trx	Thioredoxin
TF	trigger factor
GST	glutathione-S-transferase
NusA	*E. coli* transcription termination factor
SDS-PAGE	sodium dodecyl sulfate–polyacrylamide gel electrophoresis
nFel d 2	native cat serum albumin
nCan f 3	native dog serum albumin
rFel d 2	recombinant cat serum albumin
rCan f 3	recombinant dog serum albumin
dsFel d 2	recombinant cat serum albumin without N-terminal signal peptide
dsCan f 3	recombinant dog serum albumin without N-terminal signal peptide
