# Supplementary material for: A Simple Method to Produce Recombinant Mammalian Serum Albumins in Escherichia coli Preserving Intact Antigenic Properties
Source: Int J Mol Sci. 2026 May 28;27(11):4853. doi: 10.3390/ijms27114853 (PMC13256791; doi:10.3390/ijms27114853)
Supplement: Supplementary file 1 [file ijms-27-04853-s001.zip › ijms-4264658-supplementary.pdf]

Int. J. Mol. Sci.

A simple method to produce recombinant mammalian serum albumins in *E. coli* preserving intact antigenic properties

Anna S. Dolgova [1,*], Anna S. Cherkashina [2], Alexander I. Shcherbakov [2], Ksenia A. Lashkevich [2], Irina A. Goptar [3], Irina P. Lisyukova [2], Anna E. Sudina [4] and Olga A. Stukolova [2]

[1] St. Petersburg Pasteur Institute, St. Petersburg Russia, pasteur@pasteurorg.ru
[2] Central Research Institute of Epidemiology, Moscow,, Russia; crie@pcr.ru
[3] Martsinovsky Institute of Medical Parasitology, Tropical and Vector-Borne Diseases, Sechenov Univer-sity, Moscow, Russia, imp@sechenov.ru
[4] Federal State Budgetary Institution Centre for Strategic Planning and Management of Biomedical Health Risks of the Federal Medical Biological Agency, Moscow, Russia, info@cspfmba.ru
[*] Correspondence: annadolgova@inbox.ru; Tel.: +79032420614

Supplementary Material S1

Methods S1

Figure.S1 Expression and purification

Cloning

pGD vector series are based on pET24a with modified polylinker. In pGD vector in *Nhe*I/*Sac*I site are cloned different fusion tags: SlyD, SUMO, Fh8, Trx, TF, MBP, GST, NusA. Names of these vectors are pGDSlyD, pGDSUMO, pGDFh8, pGDTrx, pGDTF, pGDMBP, pGDGST, pGDNusA or pGD with no tag. The albumin genes were cut out from pGEM-T with *Nde*I/*Xho*I and ligated into the expression vector with one of the fusion tags.

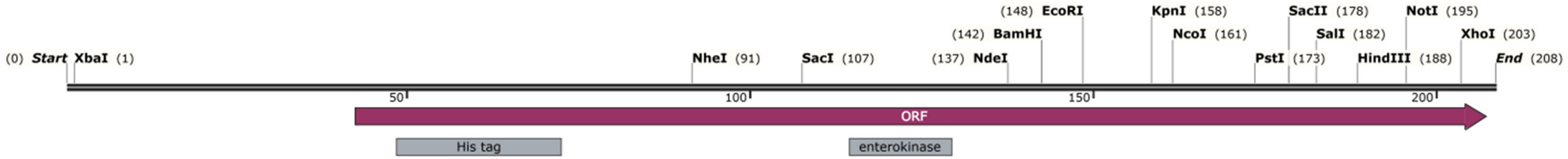

Table S1. Amino acid sequences of used tags

Tag	GenBank №	Amino acid sequence
SlyD	CAD6001205.1	mkvakdlvvslayqvrtedgvlvdespvsapldylhghgslisgletaleghevgdkfdvavgandaygqydenlvqrvpkdvfmgvdelqvgmrflaetdqgpvp veitaveddhvvvdgnhmlagqnlkfnvevvaireateeelahghvhgahdhhhdhdhdgccgghghdhghehggegccggkgnggcgch
SUMO	NP_010798.1 (Saccharomyces cerevisiae S288C)	msdsevnqeakpevkpevkpethinlkvsdgsseiffkikkttplrrlmeafakrqgkemdslrflydgiriqadqtpedldmedndiieahreqiggr AQS95516.1 (E.coli)
GST	WP_164689877.1	mspilgywkikglvqptrlllеyleekyeehlyerdegdkwrnkkfelglefpnlpyyidgdvkltqsmaiiryiadkhnmlggcpkeraeismlegavldirygvsria yskdfetlkvdflsklpemlkmfedrlchktylngdhvthpdfmlydaldvvlymdpmcldafpklvcfkkrieaipqidkylksskyiawplqgwqatfgggdhpp ksd
Fh8	AF213970_1	Mpsvqevekllhvldrngdgkvsaeelkafaddskcpldsnkikafikehdknkdgkldlkelvsilss
Trx	WP_194499549.1	msdkiihltddsfdtdvlkadgailvdfwaewcgpckmiapildeiadeyqgkltvaklnidqnpgtapkygirgiptlllfkngevaatkvgalskgqlkefldanlags
TF	WP_205899502	mqvsvettqglgrrvtitiaadsietavkselvnvakkvridgfrkgkvpmnivaqrygasvrqdvlgdlmsrnfidaiikekinpagaptyvpgeyklgedftysvefe vypevelqgleaievekpivevtdadvdgmldtlrkqqatwkekdgaveaedrvtidftgsvdgeefeggkasdfvlamgqgrmipgfedgikghkageeftidvtfp eeyhaenlkgkaakfainlkkveerelpeltaefikrfgvedgsveglraevrknmerelksairnrvksqaieglvkandidvpaalidseidvlrrqaaqrfggnekqal elprelfeeqakrrvvvglllgevirtnelkadeervkglieemasayedpkeviefysknkelmdnmrnvaleeqaveavlakakvtekettfnelmnqqa
MBP	WP_247154701.1	ssgaskieegklviwingdkgynglaevgkkfekdtgikvtvehpdkleekfpqvaatgdgpdiifwahdrfggyaqsgllaeitpdkafqdklypftwdavryngkli aypiavealsliynkdllpnppktweeipaldkelkakgksalmfnlqepyftwpliaadggyafkyengkydikdvgvdnagakagltflvdliknkhmnadtdysi aeaafnkgetamtingpwawsnidtskvnygvtvlptfkgqpskpfvgvlsaginaaspnkelakeflenylltdegleavnkdkplgavalksyeeelakdpriaatm enaqkgeimpnipqmsafwyavrtavinaasgrqtvdealkdaqt
NusA	WP_021518520.1	mnkeilavveavsnekalprekifealesalatatkkkyeqeidvrvqidrksgdfdtfrrwlvvdevtqptkeitleaaryedeslnlgdyvedqiesvtfdrittqtakqvi vqkvreaeramvvdqfrehegeiitgvvkkvnrdnisldlgnnaeavilredmlprenfrpgdrvrgvlysvrpeargaqlfvtrskpemlielfrievpeigeevieikaa ardpgsrakiavktndkridpvgacvgmrgarvqavstelggeridivlwddnpaqfvinamapadvasivvdedkhtmdiaveagnlaqaigrngqnvrlasqlsg welnvmtvddlqakhqaeahaaidtftkyldidedfatvlveegfstleelayvpmkelleiegldeptvealreraknalatiaqaqeeslgdnkpaddllnlegvdrdlaf klaargvctledlaeqgiddladiegltdekagalimaarnicwfgdea

Expression

The amount of plasmid taken for transformation, media composition and culture conditions for each variant are listed in Table 1.

Obtaining of natural albumins

Pooled animal blood plasma was obtained from The All- Russian State Center for Quality and Standardization of Veterinary Drugs and Feed. In particular, pooled cat or dog blood plasma were defreeze and centrifuged at 2500g at +4°C for 15 minutes. The proteins were precipitated by stepwise addition of solid ammonium sulfate. First ammonium sulfate were added to concentration 23% w/v followed by and after overnight incubation at +4°C and centrifugation at 10000g at +4°C

for 15 minutes. Then concentration of ammonium sulfate were increased to 33% w/v in supernatant and cold incubation and centrifugation steps were repeated. Pellet was resuspended in of 10mM TRIS buffer, pH 7.5 with 10mM NaCl, centrifuged at 10000g at +4°C for 15 minutes and obtained clean solution was loaded onto 2ml of pre-equilibrated AffiGel Blue column (BioRad, USA). Column were washed with 50mM Tris buffer, pH 8.0 with addition of 0.5M NaCl. Elution was carried out with 20 мM Na-phosphate buffer, pH 7.1, with addition of 1.5M NaCl followed with elution with 20 мM Na-phosphate buffer, pH 7.1, with addition of 2M guanidine. Obtained fractions were dialyzed against 0.1M Na-phosphate buffer, pH 7.5 and stored at +4°C.

Table S2. Conditions of recombinant protein expression

Protein	Fusion tag	MW, kDa	plasmid		Seed		Re-seed		Expression				
			V, mkl	C, ng/mkl	medium	conditions	medium	conditions	medium	Pre-induction		Post-induction	
										Duration	Conditions	Duration	Conditions
dsFeld2	no	70	2	44	Agar LB<br>50µg/ml kanamycin	Overnight,<br>+35°C	Agar LB<br>50µg/ml<br>kanamycin	Overnight,<br>+35°C	LB,<br>1% glucose<br>2% glycerol<br>50µg/ml kanamycin	3 h	+36°C<br>220rpm	4 h	+30°C<br>220rpm
	SlyD	91	2	46									
	SUMO	81	1	80	Agar LB<br>50µg/ml kanamycin<br>34µ/ml<br>Chloramphenicol<br>4% glucose				LB,<br>1% glucose<br>1% glycerol<br>50µg/ml kanamycin	2.5 h			
	Fh8	77	1	78	Agar LB<br>50µg/ml kanamycin<br>4 % glucose				LB,<br>1% glucose<br>2% glycerol<br>50µg/ml kanamycin	2 h			
	Trx	81	1	85						2.5 h			
	TF	118	1.5	50						2 h			
	MBP	110	1	63				24h<br>Room<br>temperature°	LB,<br>2,5% glycerol<br>50mM IPTG<br>50µg/ml kanamycin	Overnight	+30°C<br>220rpm	-	-
	GST	95	1	84									
	NusA	124	1	56									
dsCanf3	no	70	2	42	Agar LB<br>50µg/ml kanamycin			Overnight,<br>+35°C	LB,<br>1% glucose<br>2% glycerol<br>50µg/ml kanamycin	3.5 h	+36°C<br>220rpm	4 h	+30°C<br>220rpm
	SlyD	91	2	41									
	SUMO	81	1	68									
	Fh8	77	1,5	57						2 h			
	Trx	81	2	33						2.5 h			
	TF	119	1,5	56						2 h			
	MBP	110	1	76	Agar LB<br>50µg/ml kanamycin<br>4% glucose			24h<br>Room<br>temperature°	LB,<br>2,5% glycerol<br>50mM IPTG<br>50µg/ml kanamycin	Overnight	+30°C<br>220rpm	-	--
	GST	95	1	80									
	NusA	124	1	92									

Concentrations of recombinant proteins

Properties of the recombinant protein solutions prepared for the coating of wells of 96-well sciPLEXPLATE. Characteristics include the genes, fusion tags, the buffer they are diluted in at a predetermined concentration in mg/ml and milliliters of obtained fractions.

Table S3. Properties of recombinant proteins solutions

Protein	Fusion tag	Buffer	Concentration (mg/ml)	ml	Working concentration, µg/ml	Printing buffer
nFeld2	-	0.1M Na-phosphate buffer, pH 7.5	0.28	2	150	0.1M Na_2CO_3 pH 9,6
dsFeld2	-	50 мM Tris/HCl, pH 8.0, 0.2 M NaCl, 0.3 M imidazole, 6M Urea	1.00	10.5	100	0.5M betaine, 20% DMSO, 5% glycerol, 0.02% Tween-20 in PBS pH 7,5
dsFeld2	SlyD		2.2	11.5	200	PBS pH 7,5
dsFeld2	SUMO		0.99	14.0	200	
dsFeld2	Fh8		0.32	13.0	65	0.5M betaine, 20% DMSO, 5% glycerol, 0.02% Tween-20 in PBS pH 7,5
dsFeld2	Trx		0.38	15.0	75	
dsFeld2	TF	50 мM Tris/HCl, pH 8.0, 0.2 M NaCl, 0.3 M imidazole	1.28	15.0	200	
dsFeld2	MBP		3.76	12.0	200	
dsFeld2	GST	50 мM Tris/HCl, pH 8.0, 0.2 M NaCl, 1 M imidazole, 6M Urea	0.44	9.0	100	
dsFeld2	NusA	50 мM Tris/HCl, pH 8.0, 0.2 M NaCl, 0.3 M imidazole	1.86	9.0	200	0.1M Na_2CO_3 pH 9,6
nCanf3	-	0.1M Na-phosphate buffer, pH 7.5	0.52	2	100	0.5M betaine, 20% DMSO, 5% glycerol, 0.02% Tween-20 in PBS pH 7,5
dsCanf3	-	50 мM Tris/HCl, pH 8.0, 0.2 M NaCl, 0.3 M imidazole, 6M Urea	0.62	8.0	200	PBS pH 7,5
dsCanf3	SlyD		0.92	12.0	190	PBS pH 7,5
dsCanf3	SUMO		1.25	15.0	200	0.5M betaine, 20% DMSO, 5% glycerol, 0.02% Tween-20 in PBS pH 7,5
dsCanf3	Fh8		0.28	11.0	60	
dsCanf3	Trx		0.39	14.0	80	
dsCanf3	TF	50 мM Tris/HCl, pH 8.0, 0.2 M NaCl, 0.3 M imidazole	1.77	15.0	200	PBS pH 7,5
dsCanf3	MBP	50 мM Tris/HCl, pH 8.0, 0.2 M NaCl, 0.3 M imidazole	6.27	14.5	200	0.5M betaine, 20% DMSO, 5% glycerol, 0.02% Tween-20 in PBS pH 7,5
dsCanf3	GST	50 мM Tris/HCl, pH 8.0, 0.2 M NaCl, 0.3 M imidazole, 6M Urea	2.75	15.0	200	0.5M betaine, 20% DMSO, 5% glycerol, 0.02% Tween-20 in PBS pH 7,5
dsCanf3	NusA	50 мM Tris/HCl, pH 8.0, 0.2 M NaCl, 1 M imidazole	0.62	10.0	200	PBS pH 7,5

Methods .S2 Microarray

Protein array preparation

To measure the IgM and IgG antibody response directed against recombinant proteins, antigens and controls were spotted in triplicates using sciFLEXARRAYER SX (Scienion AG, Berlin, Germany) on the bottoms of wells of 96-well sciPLEXPLATE (Scienion AG, Berlin, Germany) to produce IFA-like tests in microarray format. As a printing negative control the buffer phosphate-buffered saline (PBS) was used. Purified Goat-antihuman IgE (Termo Fisher Scientific , USA) was used as a printing positive control series at 35µg/ml. Cystine-5 N-hydroxysuccinimide-ester labeled bovine serum albumin (BSA) (Sigma-Aldrich, Burlington, USA) was used for boarder markers and calibration. After overnight incubation at 4°C, the slides were blocked in 0.1M glycine, containing 1% Triton X-100 for 1 hour at 37°C and stored at -20°C for future use.

Protein array procedure

Just before use, wells were thawed and washed with a wash buffer (PBS with 0.05% Tween20 at pH 7.5) for 4 minutes at 37°C shaking 500rpm. After removing of wash buffer wells were incubated with serum samples diluted 1:10 in binding buffer (PBS with 1% polyvinylpyrrolidone MW 750.000 at pH 7.5) for 60 minutes at 37°C shaking 500rpm. Next, wells were aspirated, washed, and incubated with a biotin-labeled goat anti-human IgE antibodies (1:500, 2 µg/ml) (Termo Fisher Scientific , USA) in binding buffer for 30 minutes at 37°C shaking 500rpm. Wells were aspirated and washed and incubated with a Cy5-labeled streptavidin (1:500, 2 µg/ml) (Imtek, Russia) in binding buffer for 30 minutes at 37°C shaking 500rpm. Wells were aspirated and consistently washed with wash buffer and milli-Q water, and air dried at room temperature. Finally, fluorescence read out was performed by array reader Fluorowatcher (Biosciencemedia, Latvia).

Protein array read-out

Images were quantified using Fluorowatcher software (Biosciencemedia, Latvia) in accordance with the user manual. An Excel (Version 2016, Microsoft Corporation, USA) template was developed to process raw numeric data. To account for intra-test variation, medians of triplicates were calculated. Next, to eliminate background signal, the median negative control was subtracted from all tested proteins in each well. Obtained value of fluorescence for each protein were used for future calculations of cut off and conclusion of presence of antibodies to particular protein.

Table S4 Statistics

Protein	AUC	95% CI	cut off	Sensitivity	95% CI	Specificity	95% CI
nFel d2	0,580	0,518-0,640	>0	17,2	12,1 - 23,3	98,6	92,4 - 100
Fel d2_ds	0,555	0,492-0,616	>0	10,9	6,9 - 16,2	100	94,9 - 100
SlyD_Fel d2	0,568	0,505-0,628	>0	13,5	9,0 - 19,2	100	94,9 - 100
SUMO_Fel d2	0,549	0,487-0,611	>0	9,9	6,1 - 15,0	100	94,9 - 100
Fh8_Fel d2	0,542	0,479-0,603	>0	8,3	4,8 - 13,2	100	94,9 - 100
TRX_Fel d2	0,519	0,457-0,581	>0	5,2	2,5 - 9,4	98,6	92,4 - 100
TF_Fel d2	0,568	0,505-0,628	>0	13,5	9,0 - 19,2	100	94,9 - 100
MBP_Fel d2	0,576	0,513-0,636	>0	15,1	10,4 - 21,0	100	94,9 - 100
GTS_Fel d2	0,557	0,495-0,618	>0	11,5	7,3 - 16,8	100	94,9 - 100
NusA_Fel d2	0,589	0,526-0,649	>0	17,7	12,6 - 23,9	100	94,9 - 100
nCan_f3	0,578	0,513-0,640	>0	15,5	10,6 - 21,5	100	93,8 - 100
Can_f3_ds	0,532	0,468-0,596	>0	6,4	3,4 - 10,9	100	93,8 - 100
SlyD_Can_f3	0,564	0,500-0,627	>0	12,8	8,4 - 18,5	100	93,8 - 100
SUMO_Can_f3	0,537	0,473-0,601	>0	7,5	4,2 - 12,2	100	93,8 - 100
Fh8_Can_f3	0,527	0,462-0,591	>0	5,4	2,6 - 9,6	100	93,8 - 100
TRX_Can_f3	0,542	0,478-0,606	>0	11,8	7,5 - 17,3	96,6	88,1 - 99,6
TF_Can_f3	0,578	0,513-0,640	>0	15,5	10,6 - 21,5	100	93,8 - 100
MBP_Can_f3	0,570	0,505-0,633	>0	14,0	9,3 - 19,8	100	93,8 - 100
GST_Can_f3	0,535	0,470-0,599	>0	7,0	3,8 - 11,6	100	93,8 - 100
NusA_Can_f3	0,572	0,508-0,635	>0	14,4	9,7 - 20,3	100	93,8 - 100

Sensitivity and specificity with 95% CI for IgE in a serum set of IgE-positive and IgE-negative to cat or dog fur or dander samples.
Abbreviations: AUC – area under the curve, CI – confidential interval

Supplementary Material S2

dsFeld2

SlyD

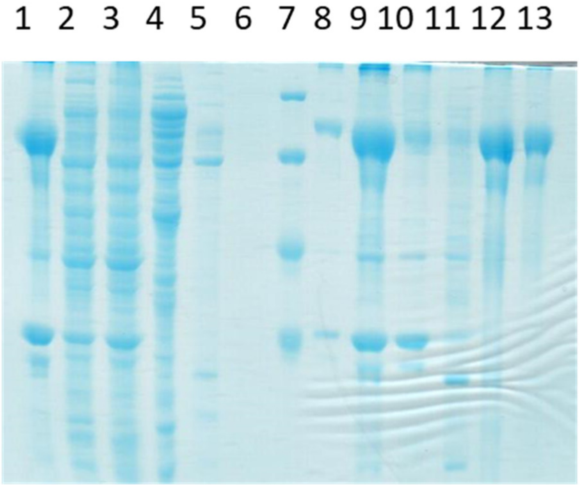

Ni-activated Chelating Sepharose chromatography
1 – soluble fraction of cell debris
2 – cell lysate
3 – flow-through of cell lysate
4 – elution with 50 мM Tris/HCl buffer (pH 8.0) with addition of 0.2 M NaCl and 0.06M of imidazole
5 – elution with 50 мM Tris/HCl buffer (pH 8.0) with addition of 0.2 M NaCl and 0.3M of imidazole
6 – elution with 50 мM Tris/HCl buffer (pH 8.0) with addition of 0.2 M NaCl and 1M of imidazole
7 – MW markers (from top: 116, 66.2, 45, 35 kDa)
8 – insoluble fraction of cell debris
9 – soluble fraction of cell debris
10 – flow-through of soluble fraction of cell debris
11 – elution with 50 мM Tris/HCl buffer (pH 8.0) with addition of 6M urea, 0.2 M NaCl and 0.06M of imidazole
12 – elution with 50 мM Tris/HCl buffer (pH 8.0) with addition of 6M urea, 0.2 M NaCl and 0.3M of imidazole
13 – elution with 50 мM Tris/HCl buffer (pH 8.0) with addition of 6M urea, 0.2 M NaCl and 1M of imidazole

SUMO

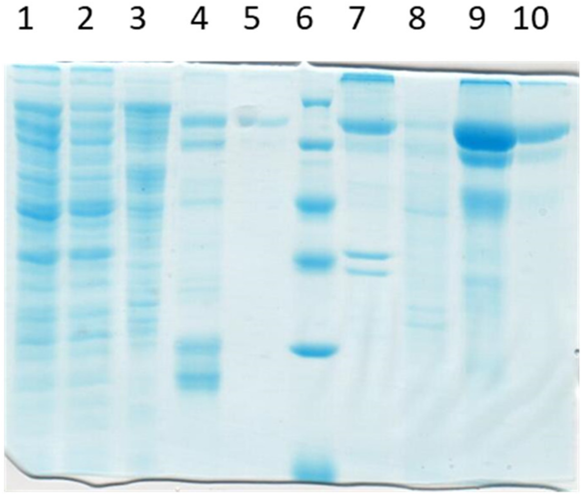

Ni-activated Chelating Sepharose chromatography
1 – cell lysate
2 – flow-through of cell lysate
3 – elution with 50 мM Tris/HCl buffer (pH 8.0) with addition of 0.2 M NaCl and 0.06M of imidazole
4 – elution with 50 мM Tris/HCl buffer (pH 8.0) with addition of 0.2 M NaCl and 0.3M of imidazole
5 – elution with 50 мM Tris/HCl buffer (pH 8.0) with addition of 0.2 M NaCl and 1M of imidazole
6 – MW markers (from top: 116, 66.2, 45, 35, 25, 18,4 kDa)
7 – flow-through of soluble fraction of cell debris
8 – elution with 50 мM Tris/HCl buffer (pH 8.0) with addition of 6M urea, 0.2 M NaCl and 0.06M of imidazole
9 – elution with 50 мM Tris/HCl buffer (pH 8.0) with addition of 6M urea, 0.2 M NaCl and 0.3M of imidazole
10 – elution with 50 мM Tris/HCl buffer (pH 8.0) with addition of 6M urea, 0.2 M NaCl and 1M of imidazole

Fh8

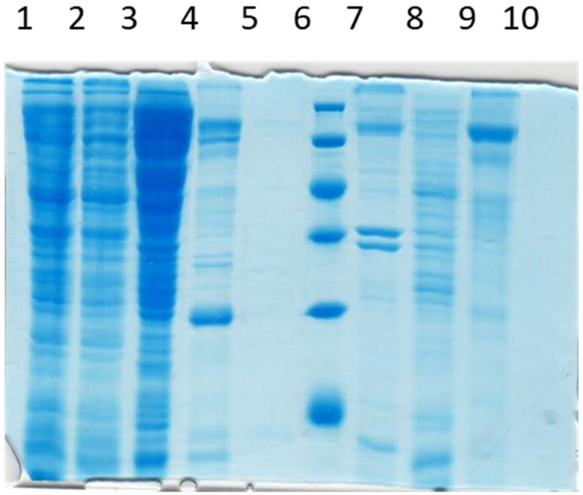

Ni-activated Chelating Sepharose chromatography
1 – cell lysate
2 – flow-through of cell lysate
3 – elution with 50 мM Tris/HCl buffer (pH 8.0) with addition of 0.2 M NaCl and 0.06M of imidazole
4 – elution with 50 мM Tris/HCl buffer (pH 8.0) with addition of 0.2 M NaCl and 0.3M of imidazole
5 – elution with 50 мM Tris/HCl buffer (pH 8.0) with addition of 0.2 M NaCl and 1M of imidazole
6 – MW markers (from top: 116, 66.2, 45, 35, 25, 18,4 kDa)
7 – flow-through of soluble fraction of cell debris
8 – elution with 50 мM Tris/HCl buffer (pH 8.0) with addition of 6M urea, 0.2 M NaCl and 0.06M of imidazole
9 – elution with 50 мM Tris/HCl buffer (pH 8.0) with addition of 6M urea, 0.2 M NaCl and 0.3M of imidazole
10 – elution with 50 мM Tris/HCl buffer (pH 8.0) with addition of 6M urea, 0.2 M NaCl and 1M of imidazole

dsFeld2

Trx

1 2 3 4 5 6 7 8 9 10

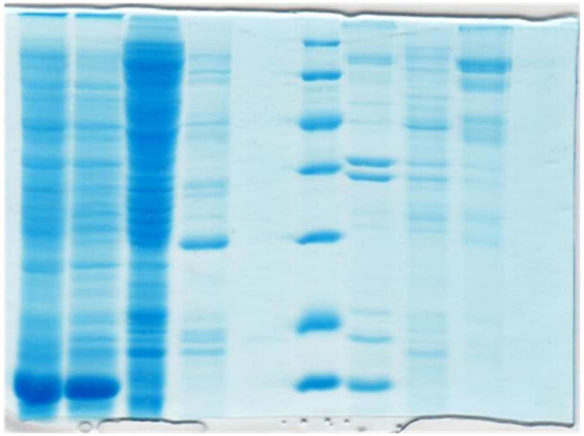

Ni-activated Chelating Sepharose chromatography
1 – cell lysate
2 – flow-through of cell lysate
3 – elution with 50 mM Tris/HCl buffer (pH 8.0) with addition of 0.2 M NaCl and 0.06M of imidazole
4 – elution with 50 мM Tris/HCl buffer (pH 8.0) with addition of 0.2 M NaCl and 0.3M of imidazole
5 – elution with 50 мM Tris/HCl buffer (pH 8.0) with addition of 0.2 M NaCl and 1M of imidazole
6 – MW markers (from top: 116, 66.2, 45, 35, 25, 18.4, 14.4 kDa)
7 – flow-through of soluble fraction of cell debris
8 – elution with 50 мM Tris/HCl buffer (pH 8.0) with addition of 6M urea, 0.2 M NaCl and 0.06M of imidazole
9 – elution with 50 мM Tris/HCl buffer (pH 8.0) with addition of 6M urea, 0.2 M NaCl and 0.3M of imidazole
10 – elution with 50 мM Tris/HCl buffer (pH 8.0) with addition of 6M urea, 0.2 M NaCl and 1M of imidazole

TF

1 2 3 4 5 6 7 8 9 10

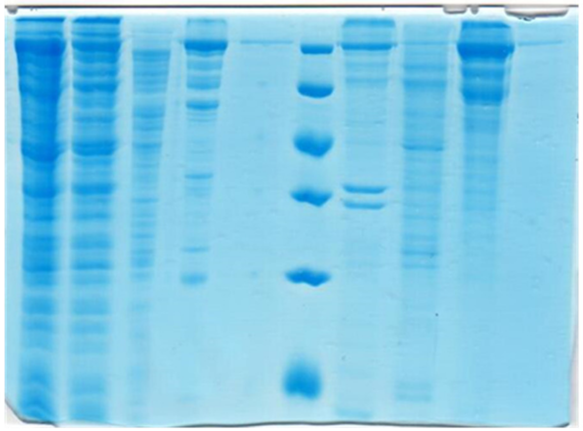

Ni-activated Chelating Sepharose chromatography
1 – cell lysate
2 – flow-through of cell lysate
3 – elution with 50 мM Tris/HCl buffer (pH 8.0) with addition of 0.2 M NaCl and 0.06M of imidazole
4 – elution with 50 мM Tris/HCl buffer (pH 8.0) with addition of 0.2 M NaCl and 0.3M of imidazole
5 – elution with 50 мM Tris/HCl buffer (pH 8.0) with addition of 0.2 M NaCl and 1M of imidazole
6 – MW markers (from top: 116, 66.2, 45, 35, 25, 18.4 kDa)
7 – flow-through of soluble fraction of cell debris
8 – elution with 50 мM Tris/HCl buffer (pH 8.0) with addition of 6M urea, 0.2 M NaCl and 0.06M of imidazole
9 – elution with 50 мM Tris/HCl buffer (pH 8.0) with addition of 6M urea, 0.2 M NaCl and 0.3M of imidazole
10 – elution with 50 мM Tris/HCl buffer (pH 8.0) with addition of 6M urea, 0.2 M NaCl and 1M of imidazole

MBP

1 2 3 4 5 6 7 8 9 10 11 12 13

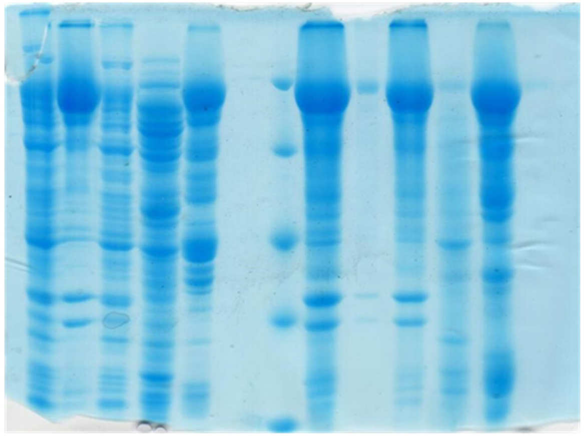

Ni-activated Chelating Sepharose chromatography
1 – cell lysate
2 – cell debris
3 – flow-through of cell lysate
4 – elution with 50 мM Tris/HCl buffer (pH 8.0) with addition of 0.2 M NaCl and 0.06M of imidazole
5 – elution with 50 мM Tris/HCl buffer (pH 8.0) with addition of 0.2 M NaCl and 0.3M of imidazole
6 – elution with 50 мM Tris/HCl buffer (pH 8.0) with addition of 0.2 M NaCl and 1M of imidazole
7 – MW markers (from top: 116, 66.2, 45, 35, 25 kDa)
8 – soluble fraction of cell debris
9 – insoluble fraction of cell debris
10 – flow-through of soluble fraction of debris
11 – elution with 50 мM Tris/HCl buffer (pH 8.0) with addition of 6M urea, 0.2 M NaCl and 0.06M of imidazole
12 – elution with 50 мM Tris/HCl buffer (pH 8.0) with addition of 6M urea, 0.2 M NaCl and 0.3M of imidazole
13 – elution with 50 мM Tris/HCl buffer (pH 8.0) with addition of 6M urea, 0.2 M NaCl and 1M of imidazole

dsFeld2

GST

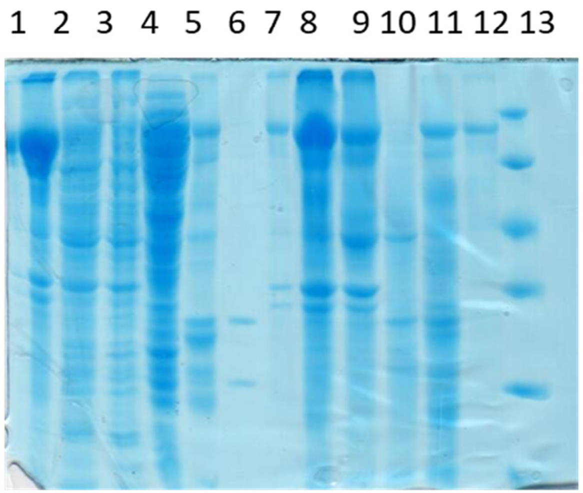

Ni-activated Chelating Sepharose chromatography
1 – cell debris
2 – cell lysate
3 – flow-through of cell lysate
4 – elution with 50 мM Tris/HCl buffer (pH 8.0) with addition of 0.2 M NaCl and 0.06M of imidazole
5 – elution with 50 мM Tris/HCl buffer (pH 8.0) with addition of 0.2 M NaCl and 0.3M of imidazole
6 – elution with 50 мM Tris/HCl buffer (pH 8.0) with addition of 0.2 M NaCl and 1M of imidazole
7 – insoluble fraction of cell debris
8 – soluble fraction of cell debris
9 – flow-through of soluble fraction of cell debris
10 – elution with 50 мM Tris/HCl buffer (pH 8.0) with addition of 6M urea, 0.2 M NaCl and 0.06M of imidazole
11 – elution with 50 мM Tris/HCl buffer (pH 8.0) with addition of 6M urea, 0.2 M NaCl and 0.3M of imidazole
12 – elution with 50 мM Tris/HCl buffer (pH 8.0) with addition of 6M urea, 0.2 M NaCl and 1M of imidazole
13 – MW markers (from top: 116, 66.2, 45, 35, 25 kDa)

NusA

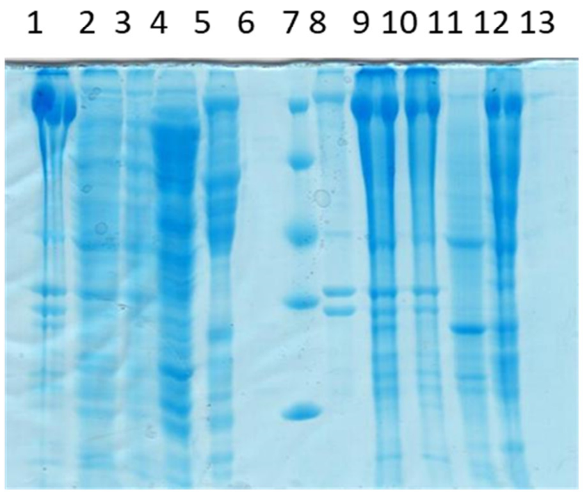

Ni-activated Chelating Sepharose chromatography
1 – cell debris
2 – cell lysate
3 – flow-through of cell lysate
4 – elution with 50 мM Tris/HCl buffer (pH 8.0) with addition of 0.2 M NaCl and 0.06M of imidazole
5 – elution with 50 мM Tris/HCl buffer (pH 8.0) with addition of 0.2 M NaCl and 0.3M of imidazole
6 – elution with 50 мM Tris/HCl buffer (pH 8.0) with addition of 0.2 M NaCl and 1M of imidazole
7 – MW markers (from top: 116, 66.2, 45, 35, 25 kDa)
8 – soluble fraction of cell debris
9 – insoluble fraction of cell debris
10 – flow-through of soluble fraction of cell debris
11 – elution with 50 мM Tris/HCl buffer (pH 8.0) with addition of 6M urea, 0.2 M NaCl and 0.06M of imidazole
12 – elution with 50 мM Tris/HCl buffer (pH 8.0) with addition of 6M urea, 0.2 M NaCl and 0.3M of imidazole
13 – elution with 50 мM Tris/HCl buffer (pH 8.0) with addition of 6M urea, 0.2 M NaCl and 1M of imidazole

Native

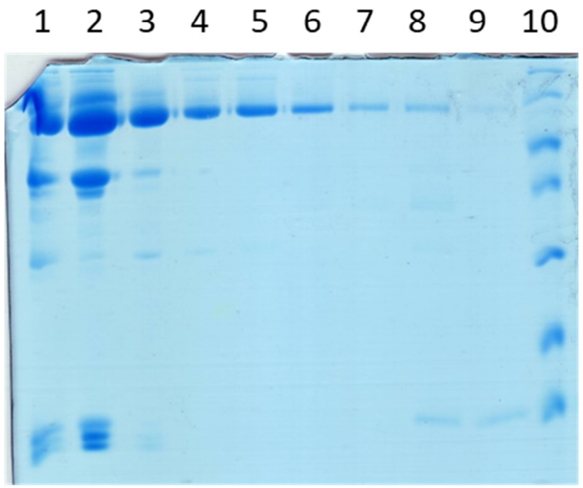

AffiGel Blue chromatography
1 – dissolved and centrifuged pellet from 33% ammonium sulfate precipitation of cat serum
2 – flow-through fraction 1
3 – flow-through fraction 2
4-5 – elution with 50mM Tris buffer, pH 8.0 with addition of 0.5M NaCl
6-7 – elution with 20 мM Na-phosphate buffer, pH 7.1, with addition of 1.5M NaCl
8-9 – elution with 20 мM Na-phosphate buffer, pH 7.1, with addition of 2M guanidine
10 – MW markers (from top: 116, 66.2, 45, 35, 25, 18.4, 14.4 kDa)

dsCanf3

SlyD

1 2 3 4 5 6 7 8 9 10 11 12 13

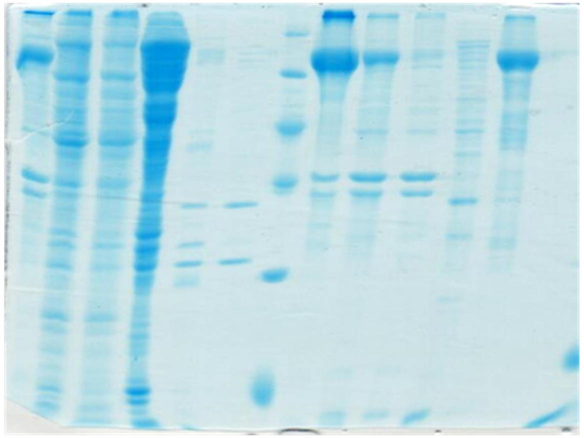

Ni-activated Chelating Sepharose chromatography
1 – cell debris
2 – cell lysate
3 – flow-through of cell lysate
4 – elution with 50 мM Tris/HCl buffer (pH 8.0) with addition of 0.2 M NaCl and 0.06M of imidazole
5 – elution with 50 мM Tris/HCl buffer (pH 8.0) with addition of 0.2 M NaCl and 0.3M of imidazole
6 – elution with 50 мM Tris/HCl buffer (pH 8.0) with addition of 0.2 M NaCl and 1M of imidazole
7 – MW markers (from top: 116, 66.2, 45, 35, 25, 18.4 kDa)
8 – soluble fraction of cell debris
9 – insoluble fraction of cell debris
10 – flow-through of soluble fraction of cell debris
11 – elution with 50 мM Tris/HCl buffer (pH 8.0) with addition of 6M urea, 0.2 M NaCl and 0.06M of imidazole
12 – elution with 50 мM Tris/HCl buffer (pH 8.0) with addition of 6M urea, 0.2 M NaCl and 0.3M of imidazole
13 – elution with 50 мM Tris/HCl buffer (pH 8.0) with addition of 6M urea, 0.2 M NaCl and 1M of imidazole

SUMO

1 2 3 4 5 6 7 8 9 10

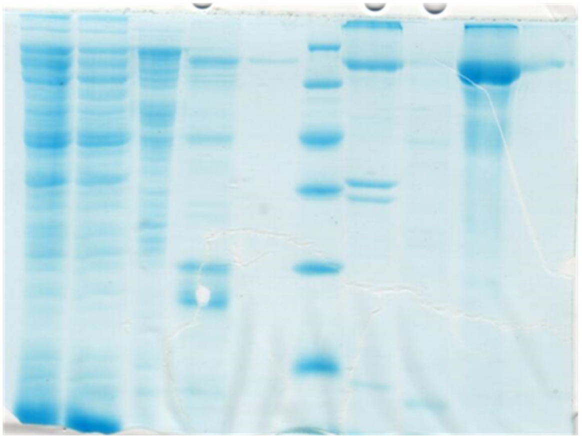

Ni-activated Chelating Sepharose chromatography
1 – cell lysate
2 – flow-through of cell lysate
3 – elution with 50 мM Tris/HCl buffer (pH 8.0) with addition of 0.2 M NaCl and 0.06M of imidazole
4 – elution with 50 мM Tris/HCl buffer (pH 8.0) with addition of 0.2 M NaCl and 0.3M of imidazole
5 – elution with 50 мM Tris/HCl buffer (pH 8.0) with addition of 0.2 M NaCl and 1M of imidazole
6 – MW markers (from top: 116, 66.2, 45, 35, 25, 18.4 kDa)
7 – flow-through of soluble fraction of cell debris
8 – elution with 50 мM Tris/HCl buffer (pH 8.0) with addition of 6M urea, 0.2 M NaCl and 0.06M of imidazole
9 – elution with 50 мM Tris/HCl buffer (pH 8.0) with addition of 6M urea, 0.2 M NaCl and 0.3M of imidazole
10 – elution with 50 мM Tris/HCl buffer (pH 8.0) with addition of 6M urea, 0.2 M NaCl and 1M of imidazole

Fh8

(compilation of 2 electrophoregrams)

1 2 3 4 5 6 7 8 9 10 11 12 13

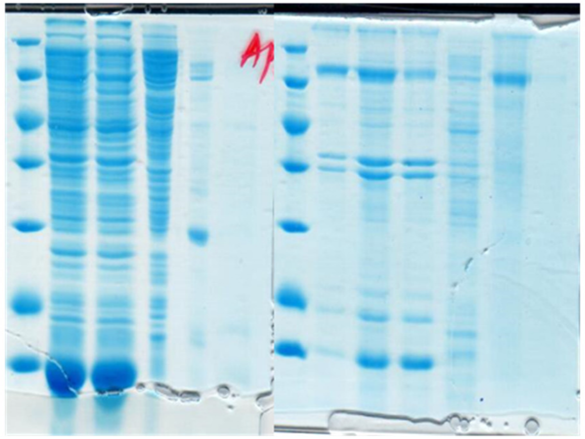

Ni-activated Chelating Sepharose chromatography
1 – MW markers
2 – cell lysate
3 – flow-through of cell lysate
4 – elution with 50 мM Tris/HCl buffer (pH 8.0) with addition of 0.2 M NaCl and 0.06M of imidazole
5 – elution with 50 мM Tris/HCl buffer (pH 8.0) with addition of 0.2 M NaCl and 0.3M of imidazole
6 – elution with 50 мM Tris/HCl buffer (pH 8.0) with addition of 0.2 M NaCl and 1M of imidazole
7 - MW markers (from top: 116, 66.2, 45, 35, 25, 18.4, 14.4 kDa)
8 – insoluble fraction of cell debris
9 – soluble fraction of cell debris
10 – flow-through of soluble fraction of cell debris
11 – elution with 50 мM Tris/HCl buffer (pH 8.0) with addition of 6M urea, 0.2 M NaCl and 0.06M of imidazole
12 – elution with 50 мM Tris/HCl buffer (pH 8.0) with addition of 6M urea, 0.2 M NaCl and 0.3M of imidazole
13 – elution with 50 мM Tris/HCl buffer (pH 8.0) with addition of 6M urea, 0.2 M NaCl and 1M of imidazole

dsCanf3

Trx

1 2 3 4 5 6 7 8 9 10

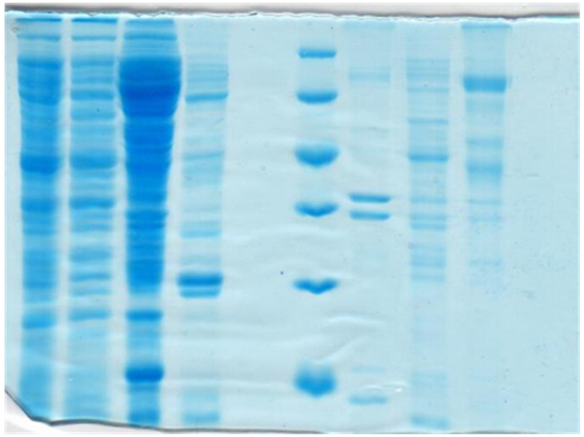

Ni-activated Chelating Sepharose chromatography
1 – cell lysate
2 – flow-through of cell lysate
3 – elution with 50 мM Tris/HCl buffer (pH 8.0) with addition of 0.2 M NaCl and 0.06M of imidazole
4 – elution with 50 мM Tris/HCl buffer (pH 8.0) with addition of 0.2 M NaCl and 0.3M of imidazole
5 – elution with 50 мM Tris/HCl buffer (pH 8.0) with addition of 0.2 M NaCl and 1M of imidazole
6 – MW markers (from top: 116, 66.2, 45, 35, 25, 18.4 kDa)
7 – flow-through of soluble fraction of cell debris
8 – elution with 50 мM Tris/HCl buffer (pH 8.0) with addition of 6M urea, 0.2 M NaCl and 0.06M of imidazole
9 – elution with 50 мM Tris/HCl buffer (pH 8.0) with addition of 6M urea, 0.2 M NaCl and 0.3M of imidazole
10 – elution with 50 мM Tris/HCl buffer (pH 8.0) with addition of 6M urea, 0.2 M NaCl and 1M of imidazole

TF

1 2 3 4 5 6 7 8 9

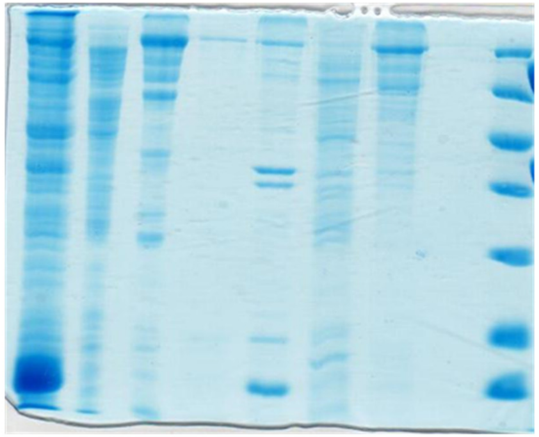

Ni-activated Chelating Sepharose chromatography
1 – flow-through of cell lysate
2 – elution with 50 мM Tris/HCl buffer (pH 8.0) with addition of 0.2 M NaCl and 0.06M of imidazole
3 – elution with 50 мM Tris/HCl buffer (pH 8.0) with addition of 0.2 M NaCl and 0.3M of imidazole
4 – elution with 50 мM Tris/HCl buffer (pH 8.0) with addition of 0.2 M NaCl and 1M of imidazole
5 – flow-through of soluble fraction of cell debris
6 – elution with 50 мM Tris/HCl buffer (pH 8.0) with addition of 6M urea, 0.2 M NaCl and 0.06M of imidazole
7 – elution with 50 мM Tris/HCl buffer (pH 8.0) with addition 6M urea, of 0.2 M NaCl and 0.3M of imidazole
8 – elution with 50 мM Tris/HCl buffer (pH 8.0) with addition of 6M urea, 0.2 M NaCl and 1M of imidazole
9 – MW markers (from top: 116, 66.2, 45, 35, 25, 18.4, 14.4 kDa)

MBP

1 2 3 4 5 6 7 8 9 10 11 12

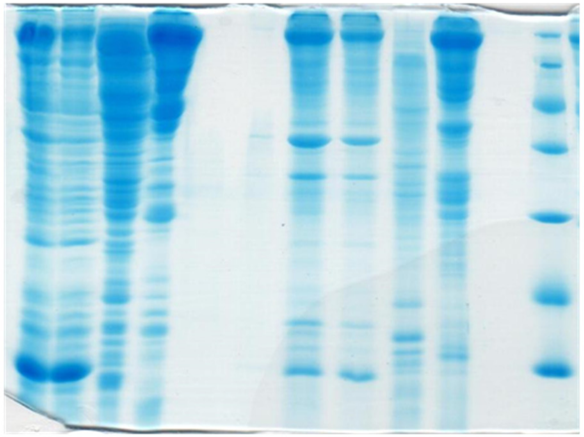

Ni-activated Chelating Sepharose chromatography
1 – cell debris
2 – cell lysate
3 – elution with 50 мM Tris/HCl buffer (pH 8.0) with addition of 0.2 M NaCl and 0.06M of imidazole
4 – elution with 50 мM Tris/HCl buffer (pH 8.0) with addition of 0.2 M NaCl and 0.3M of imidazole
5 – elution with 50 мM Tris/HCl buffer (pH 8.0) with addition of 0.2 M NaCl and 1M of imidazole
6 – insoluble fraction of cell debris
7 – soluble fraction of cell debris
8 – flow-through of soluble fraction of cell debris
9 – elution with 50 мM Tris/HCl buffer (pH 8.0) with addition of 6M urea, 0.2 M NaCl and 0.06M of imidazole
10 – elution with 50 мM Tris/HCl buffer (pH 8.0) with addition of 6M urea, 0.2 M NaCl and 0.3M of imidazole
11 – elution with 50 мM Tris/HCl buffer (pH 8.0) with addition of 6M urea, 0.2 M NaCl and 1M of imidazole
12 – MW markers (from top: 116, 66.2, 45, 35, 25, 18.4, 14.4 kDa)

dsCanf3

GST

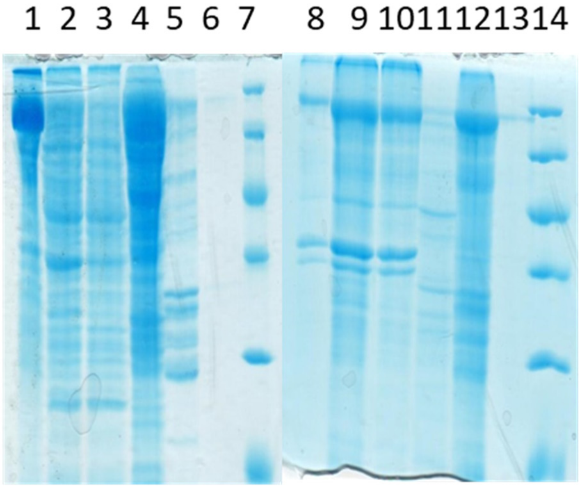

Ni-activated Chelating Sepharose chromatography
1 – cell debris
2 – cell lysate
3 – flow-through of cell lysate
4 – elution with 50 мM Tris/HCl buffer (pH 8.0) with addition of 0.2 M NaCl and 0.06M of imidazole
5 – elution with 50 мM Tris/HCl buffer (pH 8.0) with addition of 0.2 M NaCl and 0.3M of imidazole
6 – elution with 50 мM Tris/HCl buffer (pH 8.0) with addition of 0.2 M NaCl and 1M of imidazole
7 – MW markers (from top: 116, 66.2, 45, 35, 25, 18.4, kDa)
8 – insoluble fraction of cell debris
9 – soluble fraction of cell debris
10 – flow-through of soluble fraction of cell debris
11 – elution with 50 мM Tris/HCl buffer (pH 8.0) with addition of 6M urea, 0.2 M NaCl and 0.06M of imidazole
12 – elution with 50 мM Tris/HCl buffer (pH 8.0) with addition of 6M urea, 0.2 M NaCl and 0.3M of imidazole
13 – elution with 50 мM Tris/HCl buffer (pH 8.0) with addition of 6M urea, 0.2 M NaCl and 1M of imidazole
14 – MW markers (from top: 116, 66.2, 45, 35, 25, 18.4 kDa)

NusA

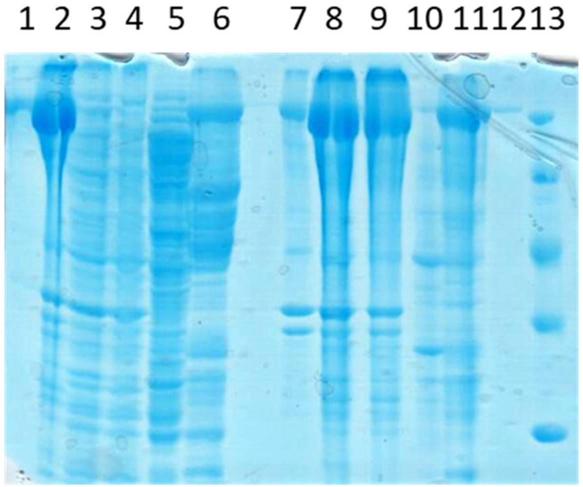

Ni-activated Chelating Sepharose chromatography
1 – cell debris
2 – cell lysate
3 – flow-through of cell lysate
4 – elution with 50 мM Tris/HCl buffer (pH 8.0) with addition of 0.2 M NaCl and 0.06M of imidazole
5 – elution with 50 мM Tris/HCl buffer (pH 8.0) with addition of 0.2 M NaCl and 0.3M of imidazole
6 – elution with 50 мM Tris/HCl buffer (pH 8.0) with addition of 0.2 M NaCl and 1M of imidazole
7 – insoluble fraction of cell debris
8 – soluble fraction of cell debris
9 – flow-through of soluble fraction of cell debris
10 – elution with 50 мM Tris/HCl buffer (pH 8.0) with addition of 6M urea, 0.2 M NaCl and 0.06M of imidazole
11 – elution with 50 мM Tris/HCl buffer (pH 8.0) with addition of 6M urea, 0.2 M NaCl and 0.3M of imidazole
12 – elution with 50 мM Tris/HCl buffer (pH 8.0) with addition of 6M urea, 0.2 M NaCl and 1M of imidazole
13 – MW markers (from top: 116, 66.2, 45, 35, 25 kDa)

Canf3 with and without signal peptide

Canf3
70,5 kDA

1 2 3 4 5 6 7 8

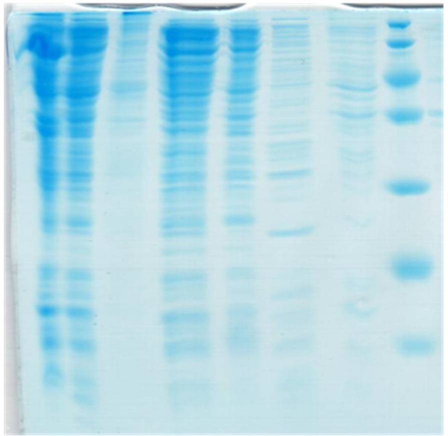

1 – cell debris
2 – cell lysate
3 – flow-through of cell lysate
4 – elution with 50 мM Tris/HCl buffer (pH 8.0)
with addition of 0.2 M NaCl and 0.06M of imidazole
5 – elution with 50 мM Tris/HCl buffer (pH 8.0)
with addition of 0.2 M NaCl and 0.3M of imidazole
6 – MW markers (from top: 116, 66.2, 45, 35, 25, 18.4, 14.4 kDa)
7 – soluble fraction of cell debris
8 – insoluble fraction of cell debris

dsCanf3
70,0 kDA

1 2 3 4 5 6 7 8 9 10 11 12 13

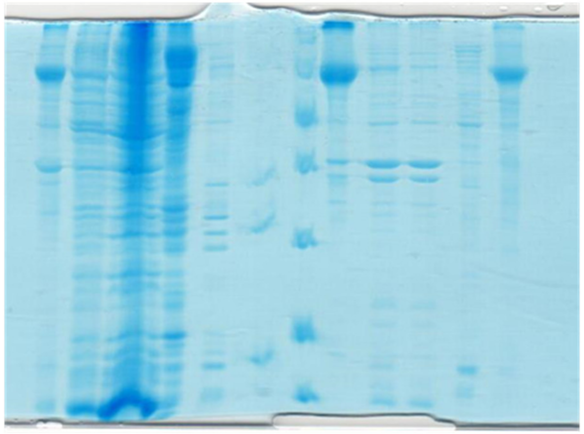

Ni-activated Chelating Sepharose chromatography
1 – cell debris
2 – cell lysate
3 – flow-through of cell lysate
4 – elution with 50 мM Tris/HCl buffer (pH 8.0)
with addition of 0.2 M NaCl and 0.06M of imidazole
5 – elution with 50 мM Tris/HCl buffer (pH 8.0)
with addition of 0.2 M NaCl and 0.3M of imidazole
6 – elution with 50 мM Tris/HCl buffer (pH 8.0)
with addition of 0.2 M NaCl and 1M of imidazole
7 – MW markers (from top: 116, 66.2, 45, 35, 25, 18.4, 14.4 kDa)
8 – insoluble fraction of cell debris
9 – soluble fraction of cell debris
10 – flow-through of soluble fraction of cell debris
11 – elution with 50 мM Tris/HCl buffer (pH 8.0)
with addition of 0.2 M NaCl and 0.06M of imidazole
12 – elution with 50 мM Tris/HCl buffer (pH 8.0)
with addition of 0.2 M NaCl and 0.3M of imidazole
13 – elution with 50 мM Tris/HCl buffer (pH 8.0)
with addition of 0.2 M NaCl and 1M of imidazole
